# Clinically Aware Learning: Ordinal Loss Improves Medical Image Classifiers

**DOI:** 10.3390/jcm15010365

**Published:** 2026-01-03

**Authors:** Arsenii Litvinov, Egor Ushakov, Sofia Senotrusova, Kirill Lukianov, Yury Markin, Liudmila Mikhailova, Evgeny Karpulevich

**Affiliations:** 1 Trusted AI Research Center, RAS, 109004 Moscow, Russia; filashkov@gmail.com (A.L.);; 2 Economic Faculty, Lomonosov Moscow State University, 119991 Moscow, Russia; 3 Higher School of Management, Financial University Under the Government of the Russian Federation, 125167 Moscow, Russia

**Keywords:** breast imaging risk classification, ordinal classification, loss functions, deep learning, breast cancer screening

## Abstract

**Background:** BI-RADS (Breast Imaging Reporting and Data System) mammogram classification is central to early breast cancer detection. Despite being an ordinal scale that reflects increasing levels of malignancy suspicion, most models treat BI-RADS as a nominal task using cross-entropy loss, thereby disregarding the inherent class order. This mismatch between the clinical severity of misclassification and the model’s optimization objective remains underexplored. **Methods:** We systematically evaluate whether incorporating ordinal-aware loss functions improves BI-RADS classification performance under controlled, architecture-fixed conditions and dataset imbalance. Using a unified training pipeline across multiple datasets, we compare ordinal losses to standard cross-entropy, analyzing the effect of dataset- and label-level balancing. Area under the receiver operating characteristic curve (AUROC) and macro-F1 scores are reported as averages over five seeds. **Results:** Balanced sampling across datasets during training led to statistically significant improvements. Ordinal loss functions, such as Earth Mover Distance (EMD), consistently achieved higher performance across multiple metrics compared to conventional cross-entropy approaches commonly reported in the literature. Improvements were particularly evident in reducing severe misclassifications, demonstrating that aligning the learning objective with the ordinal structure of BI-RADS enhances robustness and clinical relevance. **Conclusions:** Aligning the learning objective with the ordinal BI-RADS structure substantially improves classification accuracy without changing the underlying architecture. These findings emphasize the importance of loss design, regularization, and data-balancing strategies in medical AI, supporting more reliable breast cancer screening.

## 1. Introduction

Breast cancer is the second leading cause of mortality among women worldwide, despite advances in its early diagnosis and treatment [1]. Combating breast cancer remains a highly important challenge of modern medicine [2]. Timely detection of malignant changes significantly increases the chances of successful therapy [3,4]. In this regard, mammographic screening based on the interpretation of breast X-ray images plays a key role in clinical practice [5]. Breast Imaging Reporting and Data System (BI-RADS), developed by the American College of Radiology (ACR), is used worldwide to standardize the description of findings and unify clinical decision-making [6]. BI-RADS classifies detected changes by degree of suspicion for malignancy: from category 1 (no cancer) to category 6 (proven malignancy confirmed by biopsy) [7]. It is important to emphasize that this scale is ordinal and meaning-ordered, and classification errors of different magnitudes have different clinical significance [8]. At the same time, many researchers analyze mammograms using a simple binary scale: cancer/no cancer [9,10,11,12,13]. Some employ a three-class scale: benign, malignant, and normal [14,15,16], and certain studies map this tripartite scale to BI-RADS categories [17].

Experts recommend that women undergo mammography regularly starting at the age of 40 [18]. Manual analysis of mammograms is a labor-intensive and time-consuming process [19]. Automatic analysis of mammograms has become possible due to the development of deep learning methods [20,21,22]. In mammographic examinations, it is crucial to use a low radiation dose to avoid harm to patients [23]. Modern convolutional neural networks (CNNs), such as ResNet, DenseNet, and EfficientNet, help classify breast images and can improve the accuracy of screening interpretations [24]. However, most researchers treat BI-RADS classification as a standard multiclass classification task with independent categories using cross-entropy as the loss function [25]. This approach does not account for the ordinal nature of BI-RADS labels: for example, misclassifying category 3 as category 4 is far less critical than confusing categories 1 and 5, yet cross-entropy penalizes both errors equally [26]. This not only reduces interpretability but may also diminish training efficiency.

Although ordinal classification is a long-standing problem in machine learning, it has been addressed in only a limited number of studies [27]. In mammography, there are only a few attempts to apply dedicated ordinal loss functions. Some works have used multi-task architectures and correlation-based methods, but no systematic analysis of ordinal losses has been carried out [28]. In [29], different architectures were investigated for BI-RADS classification (with a custom ResNet-based network used as a building block of the model), but the loss function remained unchanged. Moreover, error analysis did not consider the degree of deviation between the predicted and true class, which is especially critical for ordinal labels.

For the publicly available INBreast dataset, many solutions report high performance with accuracy above 0.95. However, such studies often address the task as ROI-level classification rather than full image-level classification [30].

In addition, most studies focus on specific architectures and do not compare results across multiple data sources, which makes generalization of conclusions more difficult.

Automated BI-RADS classification of mammograms has become an important research problem with the development of deep learning in medical imaging. The most common approach relies on convolutional neural networks (CNNs) trained with cross-entropy loss, which treats BI-RADS categories as independent labels. This choice is largely driven by implementation simplicity and historical practice in classification tasks.

Several researchers have attempted to adapt ordinal classification methods to BI-RADS. For instance, ref. [31] applied the CORAL loss, previously successful in age estimation tasks. In [32], a complex architecture with multiple inputs and temporal slices was employed to predict malignancy, yet the loss function again did not account for ordinality.

Thus, to date, there is no comprehensive study that:

- compares modern ordinal loss functions (CORAL, CORN, CDW-CE, EMD, MCE & WK, WK) for BI-RADS classification;
- evaluates performance across multiple independent datasets reflecting different annotation practices and mammography devices;
- investigates data balancing strategies in training and their impact on performance;
- analyzes the effect of regularization techniques (e.g., label smoothing) on model quality;
- validates findings through training with multiple random seeds.

The present work addresses this gap and proposes a systematic approach to the design and analysis of BI-RADS classification models that explicitly incorporate ordinality.

In this study, we aim to fill this gap and address the following key questions:

- Can replacing cross-entropy with specialized ordinal loss functions (CORAL, CORN, CDW-CE, EMD, hybrid of MCE & WK, WK) improve the accuracy and quality of BI-RADS classification?
- How do regularization techniques, such as label smoothing, affect model performance?
- How does data balancing, given the heterogeneous size of datasets and class imbalance, influence model quality?

To this end, we conducted a series of experiments on several open BI-RADS-annotated datasets, including VinDr-Mammo, INBreast, and EMBED. We fixed the architecture (EfficientNet-B3), compared multiple loss functions and levels of label smoothing, averaged results over five random seeds, and reported macro-F1, AUROC, accuracy, macro-precision, macro-recall (sensitivity), and macro-specificity.

The main findings are as follows:

- Replacing Cross-Entropy with ordinal losses, particularly EMD and MCE & WK, leads to consistently higher AUROC and macro-F1 under a fixed architecture.
- Label smoothing does not provide meaningful improvements in validation or test performance, indicating limited usefulness of this regularization technique for BI-RADS classification.
- Dataset-level balancing, which compensates for large differences in dataset sizes, substantially improves both validation and test results.

Overall, our results highlight the importance of aligning loss functions and data balancing strategies with the medical nature of the problem, demonstrating that these design choices substantially influence model quality and generalization.

## 2. Materials and Methods

### 2.1. Datasets

To study ordinal classification behavior in mammography, we first review publicly available datasets that support severity assessment or categorical breast-lesion labeling. These datasets vary in annotation type-some include BI-RADS categories, while others provide malignancy grades or simplified labels such as normal vs. benign vs. malignant. A summary of the available datasets is presented in Table 1.

The selection criteria included the availability of formal BI-RADS assessments and the digital format of mammograms. Based on these criteria, VinDr-Mammo, INBreast, and EMBED were chosen for the main analysis, as they provide the most comprehensive and clinically relevant information.

In this study, we ensured strict patient-level separation between training, validation, and test sets, assigning all mammograms from the same patient (e.g., CC and MLO views) exclusively to either the training, validation, or test subset to avoid data leakage. VinDr includes an official split; we used only the training portion for model fitting, while the held-out VinDr portion served as the validation set for hyperparameter tuning. Since INBreast does not provide predefined splits, we created a 70/30 patient-level division and used only the INBreast test subset for final evaluation. The training set consisted of VinDr-train, INBreast-train, and the EMBED dataset.

Because the data sources (INBreast ≈ 400 images, VinDr ≈ 20 k, EMBED ≈ 300 k) differ by more than an order of magnitude in size, we adopt an evaluation protocol in which validation is performed exclusively on the held-out VinDr subset, while testing is carried out solely on the independent INBreast subset. Combining subsets during evaluation would cause the resulting metrics to be dominated by the largest source rather than reflecting overall generalization, whereas the chosen separation provides consistent model selection and a transparent estimate of cross-dataset performance.

While BI-RADS 0 represents an incomplete assessment that is not part of the diagnostic ordinal scale, the remaining categories 1–6 form an ordered system reflecting increasing likelihood of malignancy. Some datasets, such as INBreast, further subdivide category 4 into 4a, 4b, and 4c to refine risk estimation.

In this study, we addressed classification into three target categories—normal, benign, and malignant—as shown in Table 2. This mapping preserves the ordinal structure of diagnostic decisions while providing a clinically meaningful granularity aligned with practical decision-making in breast cancer screening.

BI-RADS 3, probably benign, was excluded from both the training and test sets because it represents a borderline case that typically requires short-term follow-up rather than definitive diagnosis. Including it would blur class boundaries and weaken the generalizability of the model.

We merged BI-RADS 4, 5, and 6 into a single class. Increasing the number of classes drastically reduces the number of samples available per category, which is particularly problematic for rare labels. Moreover, BI-RADS 4 varies substantially between readers and shows only moderate interobserver agreement; for non-palpable lesions with microcalcifications, it demonstrated one of the lowest reproducibility values among BI-RADS categories [41]. From a clinical perspective, BI-RADS 4, 5, and 6 all lead to the same management pathway—biopsy or definitive histopathological assessment—and thus represent a coherent “actionable” group. Although BI-RADS 5 and 6 are conceptually distinguishable, with BI-RADS 6 assigned only after biopsy confirmation, both categories indicate lesions highly suspicious for malignancy. Using three classes-normal, benign, and malignant, the latter based on merging successive ordinal BI-RADS categories-therefore still preserves the ordinal structure of the diagnostic scale.

### 2.2. Preprocessing and Augmentations

Before training, the images underwent a standard procedure of reading and preprocessing. Images with a photometric interpretation of MONOCHROME1 were automatically inverted to the MONOCHROME2 scale, in which higher pixel values correspond to brighter regions. This ensured consistency of data representation regardless of the original imaging equipment. LUT transformations, including modality and VOI LUT, were disabled, since such mappings vary between manufacturers and may introduce undesirable instability in the image dynamic range.

**Figure 1:**
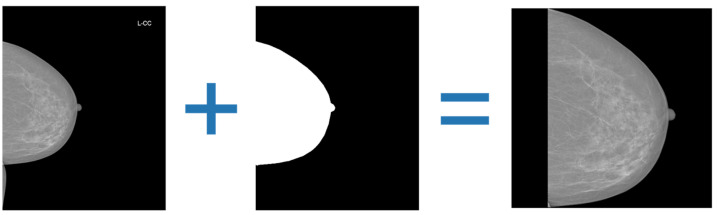
Crop example. The left image shows the original mammogram, the center shows the automatically generated binary mask of the breast region, and the right shows the resulting cropped image used for training. This step removes background artifacts and non-informative areas, ensuring consistent spatial framing and a higher proportion of diagnostically relevant breast tissue in the input image, which improves overall model prediction quality.

As a preprocessing step, breast region cropping was applied to all images (see Figure 1). Each image was associated with a binary mask delineating the breast region, generated by an external segmentation model to exclude background structures and artifacts. The breast itself was not stretched; when necessary, symmetric padding was added on both sides.

Training of models for comparing balancing strategies, loss functions, and the impact of augmentations was carried out on images with a resolution of 512 × 512 pixels. The final model was trained at a higher resolution of 1024 × 1024 pixels to better capture fine-grained diagnostic details such as microcalcifications and lesion contours. Scaling was performed to the target size while preserving the original aspect ratio, after which the images were padded to the required resolution. This procedure ensured a stable representation of anatomical structures at the model input and avoided geometric distortions that could affect classification accuracy.

A comprehensive augmentation pipeline was applied both for regularization and to increase the model’s robustness to real-world data variability. The augmentations were designed to simulate common variations in mammography-including changes in breast positioning, acquisition geometry, and illumination-while preserving diagnostically relevant content. All transformations were applied with predefined probabilities to achieve a balance between diversity and anatomical consistency.

Geometric transformations were applied to model the diversity of patient positioning and scanner geometry. Random affine transformations (*p* = 0.35) included translations up to ±6.25% of the image size, scaling in the range 0.9–1.1, and rotations within ±45°, without any shear deformation. In addition, random 90° rotations and random horizontal and vertical flips were used to introduce orientation variability. Nonlinear deformations were implemented via OpticalDistortion (*p* = 0.5, distort_limit = ±0.3) and GridDistortion (*p* = 0.2) to mimic minor geometric inconsistencies caused by compression or imaging device optics. All spatial operations employed zero padding at image borders. This set of transformations improved spatial invariance and reduced overfitting to dataset-specific acquisition geometry.

Photometric transformations simulated variability in exposure and contrast across imaging devices. These included contrast-limited adaptive histogram equalization (CLAHE, *p* = 0.35, clip_limit = 1.5), random brightness and contrast adjustments (brightness ± 0.1, contrast ± 0.2, *p* = 0.5), and gamma correction (γ ∈ (0.8, 1.2), *p* = 0.5). Image sharpening (*p* = 0.35, α ∈ (0.02, 0.2)) was also applied to enhance local contrast and improve sensitivity to microstructural details. To increase tolerance to sensor noise and small occlusions, localized dropout operations were included-PixelDropout (*p* = 0.3) and GridDropout (*p* = 0.1, ratio = 0.35). These photometric augmentations helped the model generalize across different acquisition protocols and imaging devices, while preserving the visual integrity of diagnostic features such as calcifications, masses, and architectural distortions.

This combined augmentation strategy provided a controlled yet diverse training setup that substantially reduced overfitting and improved generalization across heterogeneous datasets. It was particularly beneficial in the context of class imbalance and multi-dataset training, where robustness to acquisition and contrast variability is essential for reliable BI-RADS classification.

### 2.3. Class Balancing

In the presence of label imbalance, a model systematically shifts toward the “frequent” classes: rare observations contribute too little to the gradient and thus hardly update the parameters. As a result, performance degradation occurs precisely on the rare cases. If these rare cases correspond to clinically significant categories (e.g., cancer), this leads to reduced sensitivity for the key class and an increase in clinically dangerous misses (false negatives).

In our setting, there are two sources of imbalance:

(i) imbalance in the distribution of target labels, and
(ii) imbalance across data sources (datasets).

To address class imbalance, two main approaches can be applied: loss weighting and sample balancing.

Loss weighting involves modifying the loss function so that errors on rare examples are penalized more strongly (e.g., class- or sample-specific weights).

Advantages: (i) minimal changes to the training pipeline and code; (ii) preserves the original data distribution in batches; (iii) does not increase the number of training iterations.

Limitations: (i) not all ordinal losses correctly support weighting or allow its proper interpretation; (ii) weight tuning is sensitive to train/validation shifts and may degrade calibration; (iii) under severe imbalance, weights become extreme and amplify annotation noise.

Sample balancing refers to controlling the sampling process so that rare classes (or data sources) appear more frequently in batches, ranging from naive oversampling to stratified or uniform sampling strategies.

Advantages: (i) equalizes the contribution of rare examples to the gradient, stabilizing training; (ii) often removes the need for manual loss weight tuning; (iii) compatible with any loss, including ordinal ones.

Limitations: (i) risk of overfitting to duplicates with aggressive oversampling; (ii) increased variance of validation metrics due to mismatch with the real distribution; (iii) impaired probability calibration; (iv) requirement to fix seeds/sampling algorithms for full reproducibility.

We experimented with different combinations of balancing and evaluated their effect on validation performance. Balancing by labels ensured equal contribution of rare BI-RADS categories during training. Balancing by datasets increased the relative influence of small datasets on the overall model. It should be noted that dataset sizes differ by orders of magnitude—for example, the INBreast training set contains 267 images, whereas EMBED contains 226,717 images.

Each of the above strategies for handling imbalanced data has its own advantages and drawbacks, and the appropriate method often depends on the specific task. The results of the method comparison are presented in Section 3.1.

### 2.4. Model Architecture

All experiments were conducted using an EfficientNet-B3 model [42], pretrained on ImageNet. This architecture was chosen for its favorable trade-off between accuracy and parameter efficiency, as well as its widespread adoption in medical imaging tasks.

### 2.5. Loss Functions

It should be noted that CE treats classes as independent categories. However, many alternative losses explicitly account for the ordinal structure of classes, which is particularly suitable for problems such as distinguishing between normal, benign, and malignant cases. In this study, we aim to compare different loss functions and examine whether ordinal objectives can improve classification performance. All loss functions evaluated in our experiments are summarized in Table 3.

Implementations of CORAL and CORN were taken from the official repositories provided in the corresponding papers, while all other ordinal loss functions were obtained from [48].

### 2.6. Training

Training was carried out as a multiclass classification task using an EfficientNet-B3 convolutional neural network initialized with ImageNet-pretrained weights. The loss function and balancing strategy were systematically varied and tested, while all other training settings were kept fixed for a controlled comparison. Optimization was performed with AdamW and a step-based learning rate scheduler (see Table 4). The best model was selected according to the AUROC metric on the validation set. To ensure robustness of the evaluation, each experiment was repeated with five different random initializations, and the tables report the mean values and standard deviations of the metrics.

### 2.7. Metrics

The following metrics were used:

(1) $$Accuracy\;=\frac{Correct\;predictions}{All\;predictions}$$

For each class, we consider it as “positive” vs. all others “negative,” and define:

- True Positives (*TP*): correctly predicted positives;
- False Positives (*FP*): predicted as positive but actually negative;
- False Negatives (*FN*): predicted as negative but actually positive;
- True Negatives (*TN*): correctly predicted negatives.

Based on these counts:

(2) $$Precision=\frac{TP}{TP+FP}$$

(3) $$Recall\;(Sensitivity)=\frac{TP}{TP+FN}$$

(4) $$Specificity=\frac{TN}{TN+FP}$$

(5) $$F1=2\frac{Precision\;\cdot \;Recall}{Precision+Recall}$$

#### 2.7.1. Macro-Averaged Metrics

Each of these metrics is computed in an OVR (one-vs-rest) manner for every class and then averaged across all classes (Macro-Precision, Macro-Recall, Macro-Specificity, Macro-F1). This ensures equal weight for each class regardless of imbalance.

#### 2.7.2. AUROC (Area Under the ROC Curve)

The ROC curve plots True Positive Rate (*TPR* = Recall) against False Positive Rate (*FPR* = *FP* ÷ (*FP* + *TN*)) as the classification threshold varies.

Formally, AUROC is defined as the area under this curve:

(6) $$AUROC=\int_{0}^{1}TPR\left(FPR\right)dFPR$$

This value represents the probability that the classifier assigns a higher score to a randomly chosen positive sample than to a randomly chosen negative sample.

In the multi-class setting, AUROC is computed for each class in an OVR manner and then averaged to obtain Macro-AUROC.

### 2.8. Explainability

Explainability is widely recognized as crucial in medical AI because opaque “black-box” models can hinder clinician trust and adoption; Grad-CAM [49] is the most common technique in imaging tasks [50]. It highlights the image regions most influential for a model’s prediction, enabling insight into its decision-making process. Its successor, Grad-CAM++ [51], refines this approach using higher-order gradient weighting to produce sharper and more precise saliency maps that further enhance interpretability. In practice, these maps allow radiologists to see which tissue structures the network focuses on when assigning a BI-RADS category, helping verify that the model attends to medically relevant features. By making the decision process more transparent, such visual explanations improve human-AI collaboration - clinicians report that heatmaps increase their understanding and confidence in AI outputs.

## 3. Results

We organize the Section 3 into five themes corresponding to the main methodological components of the study. In Section 3.1, we examine the effect of data-balancing strategies, as dataset heterogeneity and class imbalance constitute major sources of variance in BI-RADS classification. In Section 3.2, we analyze the influence of label smoothing to assess whether additional regularization can enhance baseline Cross-Entropy performance. In Section 3.3, we compare loss functions, with a focus on ordinal formulations motivated by the ordered nature of BI-RADS categories. In Section 3.4, we present Grad-CAM++ visualizations for models trained with different loss functions and provide qualitative interpretability analyses. Finally, in Section 3.5, we train the model selected as optimal (EMD loss with dataset-level balancing) at higher resolution to obtain the final configuration with improved performance. The results were obtained using the services of the Shared Resource Center of the Ivannikov Institute for System Programming of the Russian Academy of Sciences – the ISP RAS Shared Resource Center.

### 3.1. Balancing vs. Loss Weighting

To assess the impact of different strategies for handling class imbalance, we performed experiments, with the results shown in Table 5 and Table 6, and statistical significance, 95% confidence intervals, and effect sizes reported in Table A1 and Table A2. Overall, using a balanced sampling strategy (oversampling/undersampling) yielded consistent gains across all metrics compared to loss weighting. The effect was especially pronounced for ordinal losses, where assigning weights correctly is not always feasible.

An important observation is the role of dataset-level balancing. The most effective strategy was dataset-level balancing combined with class-weighted cross-entropy, which achieved the highest AUROC and macro-F1 scores. Because the EMBED dataset is much larger than VinDr and INBreast, balancing by dataset reduced its dominance and prevented the model from being overly biased toward EMBED. Without this adjustment, performance on VinDr and INBreast dropped by roughly 0.04 AUROC. Balancing strategies with dataset-level balancing, both with weighted and unweighted cross-entropy, show significant improvement over the baseline in both validation and test sets, with all *p* < 0.0009.

This setup ensured a more uniform contribution of datasets while preserving the original class imbalance. While weighting improved results for cross-entropy, not all loss functions straightforwardly support weighting, and the appropriate weighting scheme may differ depending on the loss formulation. Therefore, dataset- and label-level balancing was used as a unified and unbiased configuration for fair comparison across all loss types, providing a setup that remains robust to varying degrees of imbalance and transferable across datasets. This type of balancing still outperformed the baseline by roughly 0.03 AUROC.

In our experiments, we treated data balancing and the ordinal nature of BI-RADS labels as independent factors. Thus, for a fair comparison of ordinal losses with cross-entropy and to evaluate the effect of label smoothing, we applied combined dataset- and label-level balancing, ensuring that each training set was simultaneously balanced by class labels and dataset source.

In this subsection, we focused on dataset-level balancing and demonstrated that it substantially improves performance compared to other sampling strategies. Importantly, these experiments modified only the ordering and composition of training batches, leaving the loss function unchanged. In the next subsection, we turn to the role of the loss itself and examine whether introducing mechanisms such as increased label smoothing can further enhance model performance.

### 3.2. The Influence of Label Smoothing

The influence of label smoothing on the performance of models trained with the standard Cross-Entropy loss was evaluated, with the results summarized in Table 7 and Table 8 and statistical significance reported in Table A3 and Table A4. The obtained metrics are very close across different smoothing levels, and no statistically significant improvement was observed on either the validation or the test set. This finding indicates that, under the current experimental setup and data regime, varying the label-smoothing parameter does not provide additional regularization benefits or noticeable performance gains. Consequently, these results motivate the exploration of alternative loss formulations-particularly those explicitly accounting for the ordinal structure of BI-RADS categories-to achieve further improvements in classification quality.

Since adjustments to the label-smoothing parameter within the Cross-Entropy loss do not yield meaningful performance changes, additional tuning of this loss is unlikely to provide further benefit. A more suitable direction is to investigate loss functions that explicitly model the ordinal structure of BI-RADS categories, which we examine in the next subsection.

### 3.3. Comparison of Loss Functions

The main results are shown in Table 9 and Table 10, reporting the macro-F1 and AUROC values averaged over five seed initializations for each loss function and dataset, while the corresponding statistical significance is provided in Table A5 and Table A6. Overall, ordinal losses outperform cross-entropy on average. On VinDr-Mammo (validation), EMD achieves higher AUROC and macro-F1 than CE; the AUROC gain is statistically supported (Welch’s *t*-test from summary statistics: mean, SD, *n* = 5; *p* ≈ 0.03). On the independent INBreast test, the hybrid/cost-weighted variants MCE & WK and CDW-CE deliver the strongest improvements over CE in both AUROC and macro-F1 (Welch’s *t*-test *p* ≈ 0.01 and *p* ≈ 0.04, respectively), while EMD-the best validation loss-remains above CE on average, albeit with a smaller margin. Across both datasets, macro-F1 gains for ordinal losses are directionally consistent but modest relative to between-seed variability.

On the VinDr-Mammo validation set, the use of EMD led to an increase in macro-F1 from 0.5833 to 0.5927 (+0.94 percentage points) and in AUROC from 0.7628 to 0.7694 (+0.66 pp) compared to cross-entropy. On the INBreast test set, EMD improved macro-F1 from 0.6766 to 0.6860 (+0.94 pp) and AUROC from 0.8309 to 0.8481 (+1.72 pp). These results confirm that ordinal losses are better aligned with the clinical logic of error severity than cross-entropy.

Taken together, these results suggest that the improvements are unlikely to be purely random: although the loss functions achieving statistical significance differ between the validation and test datasets, the three strongest validation losses (EMD, MCE & WK, and CDW-CE) all continue to outperform cross-entropy in AUROC on the independent INBreast test set. The distribution of significance—EMD on VinDr-Mammo and MCE & WK and CDW-CE on INBreast—reflects normal dataset-specific variability rather than inconsistency in the overall trend. Overall, the pattern indicates a consistent but modest benefit of ordinal-aware optimization, with effect sizes constrained by between-seed variation.

### 3.4. Visualizations for Model Interpretability

**Figure 2:**
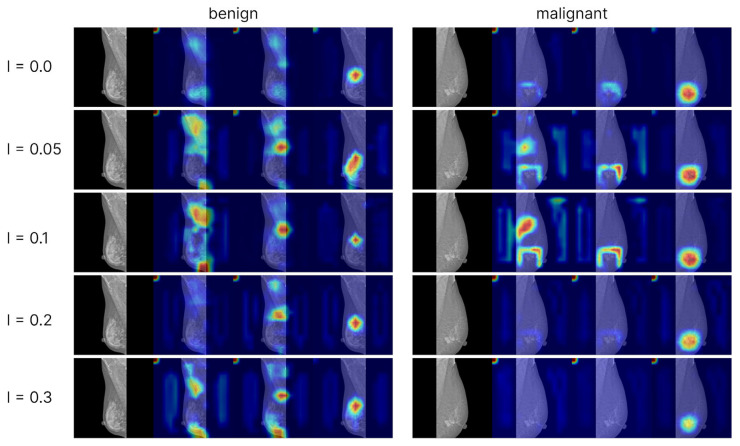
Grad-CAM++ visualizations for models trained with Cross-Entropy using different levels of label smoothing. Each example includes four columns: the original mammogram, the Grad-CAM for the normal class probability, the Grad-CAM for the benign class probability, and the Grad-CAM for the malignant class probability. The two shown mammograms correspond to correctly classified cases-the left one is benign and the right is malignant. Grad-CAM highlights the glandular regions, indicating that models focus on relevant anatomy. Variations in label smoothing do not substantially change the attention patterns, suggesting stable interpretability without overfitting.

**Figure 3:**
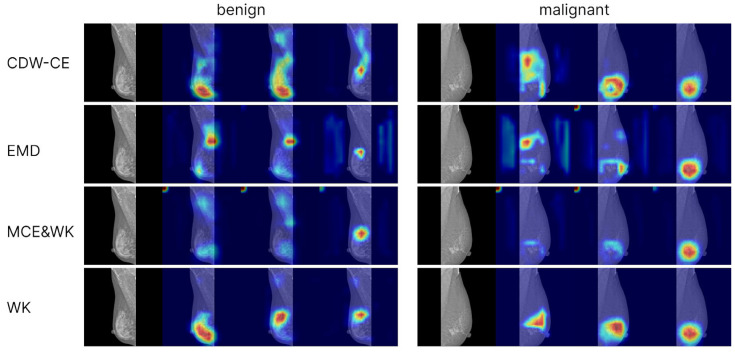
Grad-CAM++ visualizations for models trained with different ordinal loss functions. Each example includes four columns: the original mammogram, the Grad-CAM for the normal class probability, the Grad-CAM for the benign class probability, and the Grad-CAM for the malignant class probability. The two shown mammograms correspond to correctly classified cases-the left one is benign and the right is malignant. Grad-CAM shows that ordinal-loss models consistently attend to glandular tissue, supporting the clinical plausibility of learned features and robustness across loss formulations.

To analyze model interpretability, we generated Grad-CAM++ visualizations based on the convolutional network EfficientNet-B3. Activation maps from the final convolutional layer were used as the targets. Visualizations were computed for models trained with cross-entropy loss (including different levels of label smoothing) as well as for models trained with ordinal loss functions. For each class (normal, benign, malignant), separate attention maps were produced, enabling the comparison of model focus across categories and training strategies (Figure 2 and Figure 3).

The Grad-CAM++ visualizations demonstrated that the models predominantly focus on the breast region, which ensures clinical interpretability of the predictions. With label smoothing, a slight reduction in contrast was observed in the heatmaps, although the overall focus remained stable. Visualizations of models trained with ordinal losses appeared similar to those obtained with cross-entropy; in both cases, activations consistently concentrated on the relevant anatomical regions, confirming the correctness and interpretability of the models’ behavior. The areas exerting the strongest influence on the final predictions corresponded to the mammary gland itself, further confirming that the model’s decisions are driven by patient-specific breast data rather than spurious background cues, thereby indicating the absence of overfitting to non-diagnostic elements.

### 3.5. Best Model

From all experiments, we selected the best hyperparameters to train our final model. According to the balancing experiments, the most effective strategy was dataset-level balancing, while the best-performing loss function was EMD. To further improve performance, we increased the input resolution from 512 × 512 to 1024 × 1024, which resulted in an approximate AUROC gain of +0.08 on VinDr validation and +0.03 on INBreast test. These improvements are observed when comparing the final configuration with the results obtained at lower resolution in the previous stage (Table 9 and Table 10). Using this configuration, we trained the final model and obtained the results presented below (see Table 11).

## 4. Discussion

The analysis of data-balancing strategies confirmed the importance of balancing not only by class labels but also by dataset source, which helps prevent the model from overfitting to the largest dataset. In contrast, class-level balancing or applying weighted cross-entropy to rare classes had a less pronounced effect on performance. In our experiments, the best results were obtained with dataset-balanced sampling combined with weighted cross-entropy, without additional class-level balancing. These findings reinforce the importance of system-level considerations—such as data integration, fairness, and source heterogeneity—which should be explicitly addressed in real-world deployments [52,53]. The practical relevance of dataset-level balancing is underscored by the fact that medical imaging datasets often vary substantially in size, quality, and annotation practices. Our observation that equalized sampling across datasets improves generalization therefore provides a useful guideline for developing robust, context-aware training pipelines suitable for scalable and transferable AI systems in clinical settings.

The results also demonstrate that the choice of loss function has a substantial impact on BI-RADS classification accuracy, particularly on clinically meaningful metrics such as macro-F1 and AUROC. The most stable and highest-performing results were achieved with ordinal loss functions, especially EMD, across both validation and test sets. Their advantage stems from explicitly modeling the ordered structure of BI-RADS labels rather than treating categories as independent, as in the case of cross-entropy. At the same time, the observed statistical improvements were modest in magnitude and occasionally close to the boundary of detectability, reflecting the limited dataset size and between-seed variability. Nevertheless, the consistently higher average AUROC of the stronger ordinal losses across both datasets supports their practical value, particularly given the inherently ordinal nature of the clinical task. The observed benefits of ordinal-aware optimization align with broader calls for clinically meaningful AI that reflects diagnostic semantics rather than relying solely on raw accuracy metrics [54,55].

In contrast, adding label smoothing to cross-entropy did not provide statistically significant improvements. This suggests that generic regularization of the output distribution is insufficient for BI-RADS classification and that the performance gains arise specifically from incorporating the ordered structure of the labels.

Ultimately, it is essential to highlight the practical implications of these findings. Since the model architecture (EfficientNet-B3) was fixed, all observed improvements were achieved solely through the choice of loss function, balancing strategy, and regularization. This highlights that even without modifying the network architecture or increasing computational cost, substantial performance gains can be achieved through a well-founded choice of training components.

This study has several limitations. First, we excluded BI-RADS 3 because it represents a borderline category typically managed through short-term follow-up rather than definitive diagnosis, and including it would blur class boundaries and reduce model generalizability. Second, although BI-RADS is inherently multi-level, we merged categories 4–6 into a single malignant class due to limited sample sizes and the poor reproducibility of fine-grained BI-RADS labels across datasets; while clinically motivated - because BI-RADS 4 and 5 both indicate suspicion requiring biopsy and BI-RADS 6 reflects biopsy-confirmed malignancy—this reduces granularity and prevents evaluating ordinal losses on the full BI-RADS scale. Third, although the observed performance improvements of MCE & WK, EMD, and CDW-CE are statistically significant for some comparisons (EMD on validation; MCE & WK and CDW-CE on the independent test set), the effect sizes remain modest, and their magnitude should be interpreted with caution given the variability across seeds and datasets. Finally, all experiments were conducted on publicly available datasets, which do not capture the full diversity of imaging devices, acquisition protocols, or patient populations encountered in real clinical environments.

Looking forward, future research should explore how ordinal classification strategies can be extended to other medical prediction tasks that involve inherently ordered labels. Many clinical problems-such as cancer grading, diabetic retinopathy staging, fibrosis scoring, or heart failure severity classification-rely on ordinal scales that are often reduced to nominal labels in deep learning pipelines. The results presented here suggest that adopting loss functions explicitly aligned with such ordering can lead to more clinically faithful models. Additionally, the design of dataset-balancing protocols could be adapted to automated or federated learning settings, where data are distributed across institutions with heterogeneous size and label distributions. Finally, validating these methods in prospective clinical workflows and integrating interpretability tools (such as Grad-CAM++) remains essential to ensuring real-world usability and clinician trust.

## 5. Conclusions

In this study, we provide the first systematic and controlled comparison of ordinal-aware loss functions for BI-RADS mammogram classification, a task inherently defined by ordered categories but commonly treated as nominal in prior research. Our contribution lies not only in applying ordinal methods to this medical context but also in quantitatively establishing when and why ordinal alignment improves model robustness and clinical reliability under realistic constraints of data imbalance and fixed architecture.

Through a unified experimental pipeline across multiple public datasets, we isolate the effect of the learning objective itself, demonstrating that loss design, rather than architectural complexity, can drive meaningful gains in clinically relevant metrics such as AUROC and macro-F1. Importantly, our analysis reveals that ordinal-aware optimization directly mitigates severe misclassifications, aligning the model’s behavior with clinical risk interpretation, a connection that, to our knowledge, has not been systematically demonstrated before in BI-RADS classification.

By decoupling architectural factors from optimization design, this work establishes a reproducible framework for evaluating loss functions in medical ordinal tasks, bridging the gap between theoretical advances in ordinal learning and their practical application in breast cancer screening. The findings underscore that careful formulation of training objectives and dataset-level balancing strategies can yield performance and reliability improvements comparable to those achieved through far more complex model modifications.

Building on these insights, future research can explore:

- Multi-architecture ensembles that combine ordinal optimization with transformer-based or multimodal designs;
- Domain-robust training to enhance generalization across institutions and populations;
- Metadata integration (e.g., age, density, prior exams) to further contextualize ordinal predictions.

This study reframes BI-RADS classification as an ordinal optimization problem and demonstrates that aligning the learning objective with the clinical label structure is both a simple and powerful means of improving model trustworthiness. We hope these findings encourage broader adoption of ordinal principles in medical AI and inspire further work toward clinically aligned and interpretable learning frameworks.

## Figures and Tables

**Table 1:** Public datasets for mammography classification.

Dataset	Description
VinDr-Mammo [33]	A large-scale dataset that provides BI-RADS assessments at the image level, as well as local findings and breast density annotations
INBreast [34]	High-quality full-field digital mammograms annotated by experienced radiologists; includes BI-RADS categories per image and polygonal ROIs (masses, calcifications, etc.).
EMBED [35]	One of the largest US digital mammography datasets, including BI-RADS assessments, study-level metadata and diverse clinical features.
RSNA Breast Cancer Detection Challenge [36]	Contains categorical malignancy assessments at image level, but does not use the formal BI-RADS structure.
CMMD [37]	Chinese dataset with patient-level malignancy labels, without strict BI-RADS categorization.
CBIS-DDSM [38]	Updated DDSM dataset. Includes BI-RADS assessments, but images are digitized film scans rather than native digital mammograms.
MIAS [39]	UK dataset of older scanned mammograms with basic annotations not aligned to modern BI-RADS standards.
KAU [40]	Small-scale dataset with limited digital images and a reduced BI-RADS category range.

**Table 2:** Mapping of BI-RADS Categories to Model Classes. Original BI-RADS categories were merged into three target classes: normal, benign, and malignant for consistent cross-dataset training. BI-RADS 0, incomplete for diagnosis, and 3, a borderline case, were excluded.

Class Name	BI-RADS Categories
Normal	BI-RADS 1
Benign	BI-RADS 2
Malignant	BI-RADS 4 (4a, 4b, 4c), 5, 6

**Table 3:** Overview of the loss functions used for BI-RADS classification, including standard cross-entropy and multiple ordinal learning objectives.

Loss Function	Description
Cross-Entropy (CE)	Standard nominal multi-class cross-entropy; treats classes as unordered categories.
CORAL (Consistent Rank Logits) [31]	Ordinal regression via cumulative binary thresholds: transforms a (K)-class problem into (K-1) binary “is class > threshold?” subtasks, ensuring rank-consistent logits and exploiting class ordering.
CORN (Conditional Ordinal Regression for Neural Networks) [43]	Ordinal regression with conditional binary classification: solves a sequence of binary tasks conditioned on surpassing previous thresholds; recovers class probabilities via chain rule-avoids weight-sharing constraints of CORAL.
CDW-CE (Class Distance-Weighted Cross-Entropy) [44]	Weighted variant of CE: penalizes misclassifications proportionally to the ordinal distance from the true class-i.e., errors farther away are penalized more heavily, to reflect that misordering is worse when classes are far apart.
WK (Weighted Kappa Loss) [45]	Differentiable surrogate of ordinal agreement metric (e.g., quadratic or linear weighted Cohen’s kappa). This loss penalizes predictions based on how far the predicted class is from the true class on the ordinal scale; more severe ordinal errors get larger penalties.
MCE + WK (hybrid Mean Cross-Entropy + Weighted Kappa)	MCE provides stable convergence, while WK explicitly reflects the severity of ordinal errors. We use equal mixing coefficients (0.5·MCE + 0.5·WK) to balance nominal accuracy and ordinal misclassification penalties. A similar MCE + WK formulation is described in [46]
EMD (Earth Mover’s Distance/Wasserstein/Squared-EMD loss) [47]	Loss based on comparing the predicted class-probability distribution and the target (one-hot) distribution, using the Earth Mover’s Distance-which naturally takes into account the ordering of classes. In 1-D ordinal setting, often implemented as the squared EMD (equivalently squared Wasserstein-2) between cumulative distributions. This penalizes more strongly predictions that are farther away in ordinal rank.

**Table 4:** Training hyperparameters used in all experiments. Unified training settings for all models, ensuring architecture- and dataset-independent comparison of loss functions.

Parameter	Value
Model architecture	EfficientNet-B3
Pretrained weights	ImageNet
Input image size	512 × 512 (comparison: balance strategy, label smoothing, losses); 1024 × 1024 (final model).
Batch size	8
Number of epochs	100
Optimizer	AdamW
Learning rate	1 × 10^−4^
Scheduler	StepLR (γ = 0.5, step = 15,000 iterations)
Number of sampled instances per epoch	6000 (2000 from each dataset)
Metric for best epoch selection and hyperparameter comparison	AUROC on the validation set of VinDr

**Table 5:** VinDr-Mammo validation results for different balancing strategies (mean ± std over five seeds). Models using dataset-level balancing achieve the highest validation AUROC and macro-F1, clearly outperforming label-only or unbalanced approaches. This highlights the importance of controlling dataset composition during training. Bold values indicate the highest mean value for each metric.

Balancing Strategy	Accuracy	AUROC	F1	Precision	Recall	Specificity
Dataset and label balance, unweighted CE	0.7137 ± 0.0167	0.7628 ± 0.0048	0.5833 ± 0.0197	0.5702 ± 0.0268	**0.6105 ± 0.0056**	0.7967 ± 0.0045
Dataset balance, unweighted CE	0.7909 ± 0.0028	0.7832 ± 0.0026	**0.6366 ± 0.0054**	0.7637 ± 0.0157	0.5844 ± 0.0066	**0.7975 ± 0.0032**
Dataset balance, weighted CE	**0.7919 ± 0.0025**	**0.7851 ± 0.0034**	0.6333 ± 0.0074	0.7653 ± 0.0121	0.5813 ± 0.0079	0.7950 ± 0.0032
Label balance, unweighted CE	0.4910 ± 0.0391	0.7379 ± 0.0061	0.4588 ± 0.0193	0.4856 ± 0.0096	0.5535 ± 0.0144	0.7408 ± 0.0097
No balance, weighted CE	0.7111 ± 0.0272	0.7345 ± 0.0078	0.5596 ± 0.0099	0.6458 ± 0.0176	0.5290 ± 0.0137	0.7755 ± 0.0013
No balance, unweighted CE (baseline)	0.7751 ± 0.0015	0.7517 ± 0.0047	0.5644 ± 0.0068	**0.7700 ± 0.0217**	0.5150 ± 0.0059	0.7700 ± 0.0027

**Table 6:** INBreast test results: generalization of balancing strategies (mean ± std over five seeds). Models trained with dataset-level balancing show superior test AUROC and macro-F1, indicating better cross-dataset generalization and robustness to imbalance effects. Bold values indicate the highest mean value for each metric.

Balancing Strategy	Accuracy	AUROC	F1	Precision	Recall	Specificity
Dataset and label balance, unweighted CE	0.7041 ± 0.0327	0.8309 ± 0.0195	0.6766 ± 0.0263	0.6789 ± 0.0323	0.6837 ± 0.0290	0.8339 ± 0.0144
Dataset balance, unweighted CE	0.7157 ± 0.0252	0.8510 ± 0.0138	0.7131 ± 0.0260	0.7085 ± 0.0248	0.7530 ± 0.0285	0.8544 ± 0.0133
Dataset balance, weighted CE	**0.7587 ± 0.0148**	**0.8675 ± 0.0186**	**0.7486 ± 0.0173**	**0.7510 ± 0.0224**	**0.7716 ± 0.0208**	**0.8719 ± 0.0110**
Label balance, unweighted CE	0.6397 ± 0.0290	0.8169 ± 0.0130	0.6029 ± 0.0397	0.6128 ± 0.0291	0.6379 ± 0.0399	0.8193 ± 0.0124
No balance, weighted CE	0.6496 ± 0.0238	0.8100 ± 0.0084	0.6574 ± 0.0194	0.6930 ± 0.0184	0.7158 ± 0.0114	0.8325 ± 0.0089
No balance, unweighted CE (baseline)	0.6033 ± 0.0194	0.8096 ± 0.0096	0.6224 ± 0.0183	0.6854 ± 0.0146	0.6975 ± 0.0219	0.8188 ± 0.0107

**Table 7:** VinDr-Mammo validation: effect of label smoothing in Cross-Entropy loss (mean ± std over five seeds). Varying the level of label smoothing does not yield consistent improvements in validation metrics, indicating limited benefit of this regularization approach under the current training setup. Bold values indicate the highest mean value for each metric.

Smoothing	Accuracy	AUROC	F1	Precision	Recall	Specificity
0	0.7137 ± 0.0167	0.7628 ± 0.0048	0.5833 ± 0.0197	0.5702 ± 0.0268	0.6105 ± 0.0056	**0.7967 ± 0.0045**
0.05	0.7174 ± 0.0146	0.7660 ± 0.0060	0.5803 ± 0.0154	0.5681 ± 0.0199	0.6088 ± 0.0051	0.7950 ± 0.0040
0.1	0.7140 ± 0.0177	0.7647 ± 0.0042	0.5852 ± 0.0071	0.5713 ± 0.0099	0.6095 ± 0.0062	0.7955 ± 0.0058
0.2	0.7114 ± 0.0240	0.7662 ± 0.0068	0.5771 ± 0.0175	0.5634 ± 0.0248	0.6095 ± 0.0056	0.7958 ± 0.0038
0.3	**0.7273 ± 0.0115**	**0.7664 ± 0.0046**	**0.5948 ± 0.0159**	**0.5906 ± 0.0266**	**0.6115 ± 0.0082**	0.7960 ± 0.0060

**Table 8:** INBreast test: effect of label smoothing in Cross-Entropy loss (mean ± std over five seeds). No reliable gains are observed across test metrics for different label smoothing levels, confirming that label smoothing does not significantly impact generalization in this task. Bold values indicate the highest mean value for each metric.

Smoothing	Accuracy	AUROC	F1	Precision	Recall	Specificity
0	0.7041 ± 0.0327	0.8309 ± 0.0195	0.6766 ± 0.0263	0.6789 ± 0.0323	0.6837 ± 0.0290	0.8339 ± 0.0144
0.05	**0.7322 ± 0.0245**	**0.8511 ± 0.0204**	0.7056 ± 0.0419	0.7119 ± 0.0244	0.7094 ± 0.0515	0.8465 ± 0.0206
0.1	0.7174 ± 0.0465	0.8424 ± 0.0168	0.6952 ± 0.0399	0.7020 ± 0.0591	0.7017 ± 0.0373	0.8403 ± 0.0186
0.2	0.7140 ± 0.0207	0.8457 ± 0.0233	0.6897 ± 0.0276	0.6911 ± 0.0279	0.6955 ± 0.0333	0.8407 ± 0.0134
0.3	0.7306 ± 0.0290	0.8486 ± 0.0221	**0.7131 ± 0.0310**	**0.7123 ± 0.0324**	**0.7210 ± 0.0360**	**0.8470 ± 0.0149**

**Table 9:** VinDr-Mammo validation: classification performance across loss functions (mean ± std over five seeds; primary criterion AUROC). Ordinal-aware losses, particularly EMD, achieve higher AUROC and macro-F1 than cross-entropy, demonstrating the benefit of incorporating ordinal relationships in training. Bold values indicate the highest mean value for each metric.

Loss	Accuracy	AUROC	F1	Precision	Recall	Specificity
CE	0.7137 ± 0.0167	0.7628 ± 0.0048	0.5833 ± 0.0197	0.5702 ± 0.0268	0.6105 ± 0.0056	0.7967 ± 0.0045
WK	0.6512 ± 0.0222	0.7169 ± 0.0072	0.5609 ± 0.0089	0.5646 ± 0.0059	0.5685 ± 0.0103	0.7694 ± 0.0062
MCE & WK	0.6924 ± 0.0171	0.7672 ± 0.0047	0.5887 ± 0.0120	0.5839 ± 0.0142	0.5988 ± 0.0099	0.7894 ± 0.0047
EMD	**0.7257 ± 0.0196**	**0.7694 ± 0.0033**	**0.5927 ± 0.0141**	**0.5851 ± 0.0226**	0.6091 ± 0.0087	**0.7974 ± 0.0036**
CDW-CE	0.7011 ± 0.0131	0.7675 ± 0.0040	0.5916 ± 0.0076	0.5800 ± 0.0080	0.6080 ± 0.0082	0.7938 ± 0.0052
CORAL	0.7054 ± 0.0131	0.7609 ± 0.0078	0.5744 ± 0.0193	0.5570 ± 0.0203	0.6090 ± 0.0131	0.7916 ± 0.0063
CORN	0.7090 ± 0.0274	0.7592 ± 0.0045	0.5750 ± 0.0267	0.5597 ± 0.0325	**0.6145 ± 0.0049**	0.7955 ± 0.0053

**Table 10:** INBreast test: generalization of loss function performance (mean ± std over five seeds). Ordinal losses MCE & WK and CDW-CE deliver the strongest improvements, while EMD retains consistent gains over cross-entropy, confirming the generalization of ordinal-aware optimization to an independent dataset. Bold values indicate the highest mean value for each metric.

Loss	Accuracy	AUROC	F1	Precision	Recall	Specificity
CE	0.7041 ± 0.0327	0.8309 ± 0.0195	0.6766 ± 0.0263	0.6789 ± 0.0323	0.6837 ± 0.0290	0.8339 ± 0.0144
WK	0.6612 ± 0.0058	0.8135 ± 0.0154	0.6573 ± 0.0057	0.6663 ± 0.0082	0.6889 ± 0.0172	0.8238 ± 0.0095
MCE & WK	0.7471 ± 0.0245	**0.8709 ± 0.0189**	**0.7222 ± 0.0322**	0.7356 ± 0.0292	**0.7154 ± 0.0378**	**0.8503 ± 0.0172**
EMD	0.7190 ± 0.0211	0.8481 ± 0.0232	0.6860 ± 0.0233	0.6991 ± 0.0250	0.6801 ± 0.0254	0.8348 ± 0.0138
CDW-CE	**0.7488 ± 0.0171**	0.8561 ± 0.0033	0.7189 ± 0.0203	**0.7368 ± 0.0286**	0.7103 ± 0.0190	0.8500 ± 0.0049
CORAL	0.7223 ± 0.0491	0.8403 ± 0.0106	0.6984 ± 0.0423	0.7061 ± 0.0475	0.7009 ± 0.0369	0.8409 ± 0.0235
CORN	0.7124 ± 0.0343	0.8273 ± 0.0165	0.6899 ± 0.0337	0.6901 ± 0.0393	0.6950 ± 0.0280	0.8375 ± 0.0154

**Table 11:** Performance of the final model trained with EMD loss and dataset-level balancing. The results demonstrate that increasing image resolution to 1024 × 1024 substantially improves overall model quality, while maintaining the advantages of the selected loss function and balancing strategy.

Dataset	Accuracy	AUROC	F1	Precision	Recall	Specificity
VinDr (valid)	0.8246	0.852	0.6896	0.8128	0.6359	0.8444
INBreast (test)	0.7521	0.8799	0.7325	0.7301	0.7420	0.8627

## Data Availability

The data supporting the findings of this study are publicly available datasets released by their original authors. No new primary data were generated in this study. The code used for model training and evaluation is available from the corresponding author upon reasonable request.

## Abbreviations

The following abbreviations are used in this manuscript:

ACR	American College of Radiology
AUROC	Area Under the Receiver Operating Characteristic curve
BI-RADS	Breast Imaging Reporting and Data System
CDW-CE	Class Distance-Weighted Cross Entropy
CE	Cross-Entropy
CNN	Convolutional Neural Network
CORAL	Consistent Rank Logits
CORN	Conditional Ordinal Regression for Neural Networks
DL	Deep Learning
EMD	Earth Mover’s Distance Loss
MCE & WK	Mean Cross-Entropy combined with Weighted Kappa (hybrid loss)
WK	Weighted Kappa Loss

## Appendix A

This appendix complements the main results section by reproducing all six performance tables with an extended focus on statistical significance. Each table mirrors its corresponding version from the main text but augments the reported metrics with 95% confidence intervals, effect sizes (Cohen’s *d*), and *p*-values computed using a two-sided Welch’s *t*-test relative to the baseline. These additions aim to clarify whether observed differences are statistically meaningful, beyond raw mean performance comparisons.

This detailed reporting is particularly useful for readers interested in the robustness and reproducibility of the reported findings, as well as for those evaluating the validity of model comparisons. Confidence intervals provide a range of plausible values for each metric, while effect sizes and *p*-values quantify the strength and significance of differences against the baseline.

**Table A1:** VinDr-Mammo validation results for different balancing strategies (mean ± std over five seeds; see Table 5). Models using dataset-level balancing achieve the highest validation AUROC, as well as showing statistically significant improvement over the baseline.

Balancing Strategy	AUROC	95% CI	Effect Size	*p*-Value
Dataset and label balance, unweighted CE	0.7628 ± 0.0048	[0.7568, 0.7688]	2.34	0.00609
Dataset balance, unweighted CE	0.7832 ± 0.0026	[0.7800, 0.7864]	8.29	0.00001
Dataset balance, weighted CE	0.7851 ± 0.0034	[0.7809, 0.7893]	8.14	0.00000
Label balance, unweighted CE	0.7379 ± 0.0061	[0.7303, 0.7455]	−2.53	0.00445
No balance, weighted CE	0.7345 ± 0.0078	[0.7248, 0.7442]	−2.67	0.00453
No balance, unweighted CE (baseline)	0.7517 ± 0.0047	[0.7459, 0.7575]	0.00	1.00000

**Table A2:** INBreast test results for balancing strategies (mean ± std over five seeds; see Table 6). Dataset-level balancing leads to the highest test AUROC and demonstrates significant performance gains, confirming better generalization.

Balancing Strategy	AUROC	95% CI	Effect Size	*p*-Value
Dataset and label balance, unweighted CE	0.8309 ± 0.0195	[0.8067, 0.8551]	1.39	0.07225
Dataset balance, unweighted CE	0.8510 ± 0.0138	[0.8339, 0.8681]	3.48	0.00084
Dataset balance, weighted CE	0.8675 ± 0.0186	[0.8444, 0.8906]	3.91	0.00083
Label balance, unweighted CE	0.8169 ± 0.0130	[0.8008, 0.8330]	0.64	0.34450
No balance, weighted CE	0.8100 ± 0.0084	[0.7996, 0.8204]	0.04	0.94585
No balance, unweighted CE (baseline)	0.8096 ± 0.0096	[0.7977, 0.8215]	0.00	1.00000

**Table A3:** VinDr-Mammo validation: effect of label smoothing in Cross-Entropy loss (mean ± std over five seeds; see Table 7). Different levels of label smoothing do not provide significant improvement in validation performance.

Smoothing	AUROC	95% CI	Effect Size	*p*-Value
0 (baseline)	0.7628 ± 0.0048	[0.7568, 0.7688]	0.00	1.00000
0.05	0.7660 ± 0.0060	[0.7586, 0.7734]	0.59	0.38024
0.1	0.7647 ± 0.0042	[0.7595, 0.7699]	0.42	0.52440
0.2	0.7662 ± 0.0068	[0.7578, 0.7746]	0.58	0.39062
0.3	0.7664 ± 0.0046	[0.7607, 0.7721]	0.77	0.26058

**Table A4:** INBreast test: label smoothing in Cross-Entropy loss (mean ± std over five seeds; see Table 8). Consistent with validation results, label smoothing offers no significant benefit to test performance.

Smoothing	AUROC	95% CI	Effect Size	*p*-Value
0 (baseline)	0.8309 ± 0.0195	[0.8067, 0.8551]	0.00	1.00000
0.05	0.8511 ± 0.0204	[0.8258, 0.8764]	1.01	0.14822
0.1	0.8424 ± 0.0168	[0.8215, 0.8633]	0.63	0.34764
0.2	0.8457 ± 0.0233	[0.8168, 0.8746]	0.69	0.30873
0.3	0.8486 ± 0.0221	[0.8212, 0.8760]	0.85	0.21672

**Table A5:** VinDr-Mammo validation: loss function comparison (mean ± std over five seeds; see Table 9). Ordinal-aware losses, particularly EMD, yield the highest AUROC and statistically significant improvements over the baseline.

Loss	AUROC	95% CI	Effect Size	*p*-Value
CE (baseline)	0.7628 ± 0.0048	[0.7568, 0.7688]	0.00	1.00000
WK	0.7169 ± 0.0072	[0.7080, 0.7258]	−7.50	0.00001
MCE & WK	0.7672 ± 0.0047	[0.7614, 0.7730]	0.93	0.18122
EMD	0.7694 ± 0.0033	[0.7653, 0.7735]	1.60	0.03862
CDW-CE	0.7675 ± 0.0040	[0.7625, 0.7725]	1.06	0.13230
CORAL	0.7609 ± 0.0078	[0.7512, 0.7706]	−0.29	0.65753
CORN	0.7592 ± 0.0045	[0.7536, 0.7648]	−0.77	0.25610

**Table A6:** INBreast test: generalization of loss function performance (mean ± std over five seeds; see Table 10). Ordinal losses MCE & WK and CDW-CE deliver the strongest and statistically significant improvements, while EMD maintains consistent gains over cross-entropy.

Loss	AUROC	95% CI	Effect Size	*p*-Value
CE (baseline)	0.8309 ± 0.0195	[0.8067, 0.8551]	0.00	1.00000
WK	0.8135 ± 0.0154	[0.7944, 0.8326]	−0.99	0.15804
MCE & WK	0.8709 ± 0.0189	[0.8474, 0.8944]	2.08	0.01098
EMD	0.8481 ± 0.0232	[0.8193, 0.8769]	0.80	0.24112
CDW-CE	0.8561 ± 0.0033	[0.8520, 0.8602]	1.80	0.04351
CORAL	0.8403 ± 0.0106	[0.8271, 0.8535]	0.60	0.37920
CORN	0.8273 ± 0.0165	[0.8068, 0.8478]	−0.20	0.76094
